# Effects of Fermentation Process on the Antioxidant Capacity of Fruit Byproducts

**DOI:** 10.1021/acsomega.2c07602

**Published:** 2023-01-23

**Authors:** Ezgi Erskine, Gulay Ozkan, Baiyi Lu, Esra Capanoglu

**Affiliations:** † Department of Food Engineering, Faculty of Chemical and Metallurgical Engineering, Istanbul Technical University, Maslak, 34469 Istanbul, Turkey; ‡ College of Biosystems and Food Science, Zhejiang University, Yuhangtang Road 866#, Hangzhou, 310058 Zhejiang, P. R. China

## Abstract

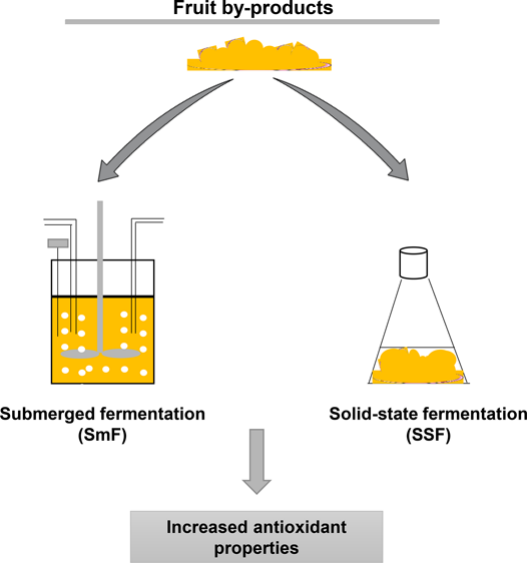

A substantial amount of fruit byproducts is lost annually due to lack of valorization applications at industrial scale, resulting in loss of valuable nutrients as well as immense economic consequences. Studies conducted clearly show that if appropriate and dependable methods are applied, there is the potential to acquire various components that are currently being obtained through synthetic manufacturing from fruit byproducts mostly regarded as waste and utilize them in not only the food industry, but pharmaceutical and cosmetic industries as well. This review aims to provide a concise summary of the recent studies regarding the fermentation of fruit byproducts and how their antioxidant activity is affected during this process.

## 1 Introduction

Food waste is unfortunately one of the biggest problems that the world is facing today, and it is constantly on the rise. An article published by the Food and Agriculture Organization of the United Nations (FAO) in 2017 states that the annual loss or waste amount of all food in the world is approximately one-third of its production, which corresponds to 1.3 billion tons of food. These losses have a big economic impact, with their annual worth equaling $990 billion. With so many countries suffering from poverty and famine, it is an enormous and unnecessary waste that the total calories lost yearly amount to roughly 24% of all food produced. Furthermore, substantial amounts of resources such as water, land, energy, and labor are completely wasted. Considering the abundance of these wastes, their negative effect on the environment is correspondingly large, and they present a crucial disposal problem. Currently there are multiple methods of disposal such as composting, incineration, animal feed production, and anaerobic digestion (AD).^1^ However, the current rate of production and corresponding disposal do not match up, and these methods remain insufficient. It has been stated that more than 60% of the wasted food can be valorized.^2^ If action is not taken now, not only will our climate, water, soil, and environment be at risk, but so will biodiversity thereby endangering sustainable food production.

Fruits and vegetables take the lead when it comes to food loss and waste, and they are responsible for 40–50% of the total waste. Large amounts of fruits and vegetables are lost during all stages, from production to consumption. Losses start during agricultural production due to factors such as climatic conditions, pests, untimely harvesting, and using incorrect or out of date harvesting methods. This step is accountable for the largest amount of loss, coming in around 5–20%. During postharvest handling and storage, insufficient and unhygienic transportation, failing to maintain the cold chain, and damaging the produce during loading and dumping likewise result in a significant amount of loss. Processing and packaging steps also have a number of difficulties including improper maturation practices, unsterile and inadequate surroundings, using improper produce and packaging material, and many more. During distribution and consumption, the biggest losses result from improper storage conditions and physical damage; this accounts for 0.5–10% of total loss.^3^ Another factor in food waste during the consumption stage is the large emphasis on the appearance of products. Perfectly fresh fruits and vegetables are thrown away in big masses due to their displeasing physical aspects.

One of the important losses, if not the most important, to take into consideration which applies not only to fresh products but also to nearly all kinds of processed foods is the byproducts. Ayala-Zavala et al.^4^ explained that fruit byproducts can be made up of more than 50% of the fresh fruit itself as they contain bagasse, peels, trimmings, stems, shells, bran, and seeds. It has been further explained that these byproducts are known to be incredible sources of valuable compounds including proteins, lipids, starch, micronutrients, bioactive compounds, and dietary fibers. However, there are certain limitations on the use of these byproducts in the food industry as a result of the antinutritional factors (ANF) they contain. Some of these are condensed tannins, saponins, trypsin inhibitors, phytates, and isoflavonoids.^5^ ANF are known to negatively affect the digestibility and bioavailability of proteins, carbohydrates, and minerals. Accordingly, there are various studies regarding the treatment of ANF with fermentation being one of the more efficient processes in this regard.^6^

The physicochemical and biochemical alterations that occur during fermentation have been shown to have beneficial outcomes such as bringing about an increase in the protein, essential amino acids, essential fatty acids, and vitamin contents. Additionally, the digestibility of the food is increased, and the production of antimicrobial compounds results in an extended shelf life.^7^ Although submerged fermentation (SmF) is generally more utilized, solid state fermentation (SSF) is a fermentation process which requires the presence of an infinitesimal amount of water and is increasingly popularizing due to the various advantages it carries in contrast to SmF.^8^

The amount of usable products that is lost and discarded as waste on a daily basis should not be undermined. Even though completely eliminating these losses is nearly impossible, they can be minimized drastically through training, creating awareness and improving the motivation and skills of the employees involved in these steps. A great deal of these losses are perfectly fresh and edible produce or byproducts, which could be consumed, processed, or utilized for the components they possess.^9^ It is crucial to establish an awareness of the importance of both the reduction of food waste resulting in each step, from harvest to consumption, and also the recycling of these foods. Humans have still not realized the extent of the potential that could be obtained from plants. The majority of these recyclable wastes and byproducts that are thrown away, along with the components they contain, could be a huge asset to the food industry. As mentioned above, even the remains of all types plants, not only fruits and vegetables but also seeds, nuts, grains, and legumes, are incredible sources of a variety of nutrients that could be further enhanced through fermentation and utilized in a number of ways in the food, cosmetics, and pharmaceutical industries.

Another factor worth contemplating is that consumers are becoming more conscious each day regarding their diet. They are trying to avoid synthetic preservatives and certain types of processed foods as much as they can.^4^ As a consequence, the food industry is continuously trying to find alternative additives that are natural in order to provide healthier products and please the public. Recycling the wastes and byproducts of fruits and vegetables is a method that offers a solution to these problems. These products already contain colorants, flavorings, and other constituents that provide antimicrobial and antioxidant properties naturally and are able to extend the shelf life of products in a safe way without the use of synthetic components.

It has been demonstrated in numerous studies that the fermentation process has the ability to increase certain valuable components present in agrowastes and therefore enhance the yield of compounds with antioxidant properties. If appropriate and dependable extraction methods are applied, this could result in acquiring various components that are currently being obtained through synthetic manufacturing. Taking into account the health benefits of plants in general, it can also be deduced that the components obtained from their infinite variety would also have a wide range of advantageous uses in the pharmaceutical and cosmetic companies as well.^4,10^ Considering the explanations above, this review aims to provide an overview of the recent applications of SmF or SSF on various fruit byproducts for the purpose of obtaining natural components with antioxidant properties. There is affluent research in the literature with respect to using fermentation on fruit byproducts to obtain a number of different compounds such as antimicrobials, colorants, peptides, and enzymes, along with studies which observe the effect of fermentation on a diverse range of nonwaste foods. This review primarily focused on the food industry research and fermentation of fruit byproducts conducted in the last 10 years. Furthermore, commercial applications and customer acceptance were briefly discussed, along with the importance of applying this technology on agroindustrial wastes in abundance in individual countries.

## 2 Natural Antioxidants

Natural antioxidants are plentiful in nature, found in both food and medicinal plants. Studies concerning antioxidants are generally in agreement regarding their benefits on human health such as anti-inflammatory, antibacterial, antiviral, antiaging, and anticancer effects.^11^ The principal reason behind these advantages is due to the components’ abilities to counterbalance problems caused by oxidative stress in the human body. Oxidative stress is a result of the inability of the endogenous antioxidants in our body to equalize the attacks of reactive oxygen species (ROS).^12^ This is attributed to a variety of factors such as air pollution, cigarette smoke, alcohol intake, and radiation and bacterial, fungal, or viral infections.^13,14^ When used in food products, antioxidants are able to stabilize lipids, inhibit oxidation, and extend shelf life, thereby improving the product’s overall quality. For a long time, synthetic chemicals such as butylated hydroxyanisole (BHA), butylated hydroxytoluene (BHT), propyl gallate (PG), and *tert*-butylhydroquinone (TBHQ) were used in foods.^15^ These were preferred by manufacturers as they were cost-effective, simpler to access, more stable in terms of quality, and had enhanced antioxidant properties.^16^ However, as a result of consumer demands for natural alternatives along with various reports challenging the safety of the widely used synthetic antioxidants, researchers shifted their attention toward studying natural antioxidants as well as their enhancement, extraction, and application.^17^ Components exhibiting antioxidant activity can be classified in two categories: hydrosoluble and liposoluble. Hydrosoluble compounds are mainly polyphenols, while the liposoluble ones are carotenoids.^11^

In nature, over 8000 molecules with a polyphenol structure have been identified, with the main classes being phenolic acids, flavonoids, lignans, and stilbenes.^18^ They are compounds that are found in plant food sources such as grape seeds, apples, pomegranate, green tea, cranberries, beetroot, and potato, among others. It has also been demonstrated that there are up to 200–300 mg of polyphenols per 100 g of fresh weight of fruits such as grapes, apples, etc., while there are 100 mg of polyphenols in a glass of red wine or a cup of tea. Mainly skins, pulps, and seeds of grapes contain phenolic compounds since those parts are partially extracted during wine production. Polyphenols are used in the treatment of diabetes, cancer, and cardiovascular diseases because of their high antioxidant and anti-inflammatory properties. Amount of consumption of polyphenols and their bioavailability determine the health effects of polyphenols.^18^ On the other hand, carotenoids are natural fat-soluble pigments that give red, orange, and yellow color to plant leaves, fruits, vegetables, and flowers.^19^ Over 700 compounds of carotenoid nature have been discovered to date; however, only 50 of these can be metabolized by humans. The main carotenoids used in the food, pharmaceutical, cosmetics, and animal feed industries, predominantly as colorants, are α-carotene, β-carotene, lycopene, lutein, and zeaxanthin.^20^ However, as they are also effective singlet oxygen quenchers, they are utilized as antioxidants in the same industries against cancer, cardiovascular, and photosensitivity disorders.^21^ Yellow-orange fruits contain notable amounts of α-carotene and β-carotene, while orange fruits are known to have α-cryptoxanthin and zeinoxanthin.^22^

## 3 Effect of Fermentation on Antioxidant Activity

Fermentation is an important part of food biotechnology which has been around since Neolithic times.^23^ Initially, it was used to preserve food products and prevent diseases; the process evolved with human civilization to the point that now we are able to use it to produce and extract bioactive compounds.^24^ Fermentation is an anaerobic process where molecules such as sugars are broken down via various microorganisms and enzymes to be converted into alcohols. This can be achieved by two methods, namely, SmF and SSF. The principle of SmF is that a liquid medium is required as a source of nourishment for the microorganisms in order for fermentation to take place.^25^ In contrast, SSF is the same process in the absence or near absence of free water in the medium, where the substrate itself is required to contain the necessary moisture to allow the microorganism to survive and grow.^26^

SmF is the more widely applied method of the two, mainly as a result of certain advantages it possesses over SSF. The most noteworthy of these are better process control and handling, along with easier heat transfer.^27^ By nature, SmF is more suitable for the processing of liquid wastes, and its implementation is especially common in large-scale production of enzymes and other bioproducts.^28^ However, there are claims of SSF, rather than SmF, being a more suitable candidate for commercial enzyme production due to the higher yield achieved when the two processes were compared using the same strain.^29^ Although not as conventional as SmF, SSF has been sparking the interest of both the commercial and science worlds in recent years. As it is a solid-state fermentation, it has considerably lower water and energy requirements, which consequently lowers bacterial contamination along with sterilization and production costs.^30^ The system of SSF also involves a smaller fermenter size along with an increase of product yield and quality, gradually becoming preferred to SmF. This is especially applicable for solid waste management as SSF enables the utilization of these wastes without pretreatment.^31^ However, the current detriments of SSF are mainly due to heterogeneities regarding heat and mass transfer due to the absence of excess water. Another limitation is the fact that SSF is a difficult process to actualize on an industrial scale. The main reasons behind this are the aforementioned complications regarding heat and mass transfer homogeneity. However, it must be kept in mind that SSF is not as widely researched as SmF, making its process optimization and industrial application an area with gaps.^31^ Studies which compare the two fermentation processes generally state that the complications of SSF could be minimized through further research and that the process itself is worth looking into further.

Setting up an SSF system requires the careful selection and control of a variety of parameters, as managing the process conditions of this type of fermentation is extremely challenging even at small scales.^32^ Suitable microorganism strains which will be able to both produce the desired bioactive compounds and carry out the fermentation process in solid state conditions must be chosen.^24^ The type of bioreactor is another crucial factor to consider when setting up an SSF system. By nature, this type of fermentation has very little to no water; thus, there is a limitation in terms of heat transfer and thermal conductivity. There have been attempts to improve this by the gas phase between the particles. Although the thermal conductivity of the gas phase is considerably lower in comparison to water as in SmF, it is still an important characteristic of SSF. The efficiency of the SSF process is effected by numerous factors with some of the most crucial ones being pretreatment and particle size of substrates, medium ingredients, supplementation of growth medium, sterilization of SSF medium, selection of microorganism, moisture content, water activity (aw), inoculum density, temperature, pH, agitation, and aeration.^33^ When it comes to setting up an SmF system, molasses, fruit and vegetable juices, and sewage/wastewater are the prevalent liquid substrates required for the process.^34^ One of the most critical aspects of the bioreactor used in SmF is its aeration system as it can directly have an effect on oxygen transfer, temperature control, and achieving homogenization.^34^ Heating and cooling systems are also utilized to prevent temperature fluctuations leading to fermentation breakdown. Aeration is another contributing factor to the system setup, influencing the oxygen absorption of the microorganisms.^35^

Antioxidant activity is due to the protective activity of a large variety of compounds against oxidation. Although these compounds can be found in various food products, their bioaccessibility may be limited. Verni et al.^10^ reported that these compounds can be released or converted into more active forms through fermentation and attribute these changes to metabolic activities affecting phenolic acids, flavonoids and tannins, the release of antioxidant peptides, changes in vitamin contents, and the production of exopolysaccharides.^10^ The main reason behind the positive correlation between fermentation and antioxidant activity has been predominantly attributed to microbial hydrolysis, which causes the quantity of phenolic compounds and flavonoids in the food to increase. Furthermore, various aforementioned antioxidant compounds are liberated or synthesized due to the structural breakdown of plant cell walls. A majority of these compounds are able to act as free radical terminators, metal chelators, singlet oxygen quenchers, or hydrogen donors to radicals.^36^ Another contributing factor may be that the lactic acid bacteria that are present during fermentation have antioxidant activity. Studies showcase the advantages of lactic acid fermentation and its effect on improving the functional properties of certain food products.^37^ It was stated that phenolics can be produced by the microorganisms present during fermentation or released from the substrate.^38^ The mechanisms of fermentation which have a positive effect on antioxidant activity are multifarious; thus, further research is required to determine these methods more definitively. Considering the high numbers of studies which indicate that fermentation improves antioxidative activity, it is an area that is worth investigating in detail as the process is important in terms of obtaining natural antioxidants.

Studies analyzing the effects of fermentation type and conditions on the antioxidant properties of various fruit byproducts are summarized in Table 1.

**Table 1:** Studies on the Effects of Fermentation Type and Conditions on the Antioxidant Properties of Various Fruit Byproducts^a^

fruit byproduct	microorganism	outcome	fermentation type	fermentation conditions	phenolic extraction solvent	country	ref
pineapple	*Kluyveromyces marxianus* NRRL Y-8281	TPC↑, FRSA↑, AO↑, RA↑	SSF	3 days at 30 °C	methanol	Egypt	(39)
pineapple residue and soy flour	*Rhizopus oligosporus*	TPC↑, FRSA↑	SSF	12 days at 20–22 °C	water/ethanol	Brazil	(40)
pomegranate peels and soy flour	*Aspergillus niger*	TPC↑, FRSA↑, AO↑	SSF	4 days at 28 °C	water	India	(41)
grape pomace	*Actinomucor elegans*	TPC/TFC↑ until day 4, TPC/TFC↓ after day 4	SSF	12 days at 28 °C	hydrochloric acid/methanol/water	Romania	(42)
	*Umbelopsis isabellina*	TPC↓, TFC↓	SSF	12 days at 28 °C	hydrochloric acid/methanol/water		(42)
grape waste, olive oil waste, beer waste	*Rhizopus oryzae*	TPC↑, FRSA↑, AO↑	SSF	7 days at 25 °C	water and lignocellulolytic enzyme	Portugal	(43)
black grape, apple and yellow pitahaya residues	*Rhizomucor miehei* NRRL 5282	TPC↑, AO↑	SSF	18 days at 37 °C	water and water/ethanol	Hungary	(44)
grape waste	*Aspergillus niger* GH1, PSH, Aa-20, and ESH	TPC↑, FRSA↑, AO↑	SSF	60 h at 30 °C	water	Mexico	(45)
mango seeds	*Aspergillus niger* GH1	TPC↑, FRSA↑, AO↑	SSF	60 h at 30 °C	ethanol		(6)
fig byproducts	*Rhizopus oryzae* (PP4-UAMI)	TPC↑	SSF	72 h at 30 °C	citrate buffer		(46)
	*Trichoderma* sp.	FRSA↑	SSF	72 h at 30 °C	citrate buffer		(46)
	*Aspergillus niger* HT4	AO↑	SSF	72 h at 30 °C	citrate buffer		(46)
	*A. niger* GH1	AO↑	SSF	72 h at 30 °C	citrate buffer		(46)
grapefruit	*Aspergillus niger* GH1	70% moisture, AO↓ until 24 h, AO↑ after 24 h; 50% moisture, AO↑	SSF	120 h at 30 °C	ethanol		(47)
plum pomace	*Aspergillus niger*	TPC↑, TFC↑	SSF	14 days at 30 °C	hydrochloric acid/methanol/water	Romania	(48)
	*Rhizopus oligosporus*	FRSA↑, AO↑	SSF	14 days at 30 °C	hydrochloric acid/methanol/water		(48)
apricot pomace	*Aspergillus niger*	TPC↑, TFC↑	SSF	14 days at 30 °C	hydrochloric acid/methanol/water		(49)
	*Rhizopus oligosporus*	FRSA↑, AO↑	SSF	14 days at 30 °C	hydrochloric acid/methanol/water		(49)
chokeberry pomace	*Aspergillus niger*	TPC↑, TFC↑	SSF	12 days at 30 °C	hydrochloric acid/methanol/water		(50)
	*Rhizopus oligosporus*	FRSA↑, AO↑	SSF	12 days at 30 °C	hydrochloric acid/methanol/water		(50)
cranberry pomace	*Rhizopus oligosporus*	TPC↑, FRSA↑, AO↑	SSF	16 days at 28 °C	ethanol	United States of America	(51)
apple pomace	*Phanerocheate chrysosporium*	TPC↑, FRSA↑, AO↑	SSF	10 days at 37 °C, pH 4	acetone or ethanol	Canada	(52)
cocoa pod husk, cassava peel and palm kernel	*Rhizopus stolonifer LAU* 07	FRSA↑, AO↑	SSF	5 days at 30 °,C pH 6.4–6.7	methanol	Nigeria	(53)
cocoa meal	*Penicillium roqueforti*	TPC↑, FRSA↑, AO↑, RA↑	SSF	7 days at 25 °C	water and hydroethanol	Brazil	(54)
orange pomace	*Paecilomyces variotii*	TPC↓, AO↑	SSF	120 h at 30 °C, 90% relative humidity	aqueous acetone		(55)
orange, carrot, and papaya peels	*Blakeslea trispora* (+) MTCC 884	TPC↑, FRSA↑, AO↑	SSF	90 days at 25–32 °C, pH 6–7	petroleum ether	India	(56)
acerola (*Malpighia emarginata DC.*)	*Lactobacillus* isolates: *L. casei* L-26, *L. fermentum 56*, *L. paracasei* 106, and *L. plantarum* 53	TPC↑	SmF	120 h at 37 °C	methanol	Brazil	(57)
guava (*Psidium guajava L.*)	*Lactobacillus* isolates: *L. casei* L-26, *L. fermentum 56*, *L. paracasei* 106, and *L. plantarum* 53	AO↑	SmF	120 h at 37 °C	methanol		(57)
blueberry	*L. rhamnosus GG*, *L. plantarum*-1, and *L. plantarum*-2	TPC↑, AO↑	SmF	28 h at 37 °C, pH 6.2	ethanol	China	(58)
pomelo peel	F33 French active dry wine yeast	TFC↑; 9.4% concentration, AO↑	SmF	fermentation, 10 days at 25 °C; postfermentation, 20 days at room temperature	alcohol		(59)

a TPC, total phenolic content; TFC, total flavonoid content; FRSA, free-radical scavenging activity; AO, antioxidant activity; RA, reducing activity; CA, chelating activity; SmF, submerged fermentation; SSF, solid state fermentation; ↑, value higher than nonfermented sample; ↓, value lower than nonfermented sample/difference can be neglected.

According to FAO, approximately half of the total pineapple is discarded during canning or consuming, which unfortunately results in around ten tons of fresh pineapple waste per hectare. In a study conducted by Rashad et al.,^39^ unfermented and SSF fermented pineapple waste extracts at different concentrations were compared in terms of their total phenolic content, DPPH free radical scavenging effects, antioxidant activity, reducing effects, chelating ability, and *in vitro* anticancer activity against different human cancer cell lines such as liver HepG2, breast MCF-7, lung A549, acute myeloid leukemia HL-60, and colon HCT116. Pineapple residue consisting of pulp, peels, skin, core, and crown was collected from a juice extraction shop and analyzed to observe the chemical changes occurring during SSF with the microorganism *K. marxianus* NRRL Y-8281. The fermented pineapple waste showed higher results in all areas of interest compared to the unfermented extracts. The highest phenolic content was recorded to be 120 mg GAE/100 g dry weight (dw) at a concentration of 8 mg/mL, with the totals being inversely proportionate to concentrations above this value. The results also showed an increase in the linoleic acid radical scavenging activity of pineapple waste, with the unfermented activity being 88% and fermented 95%. In terms of anticancer activity, both fermented and unfermented extracts exhibited effects close to the doxorubicin drug against MCF-7, A549, and HCT116 cell lines, although the fermented samples were shown to be much more effective than their nonfermented counterparts. Furthermore, gas chromatographic/mass spectrometric (GC/MS) analysis was conducted to identify the compounds believed to be responsible for said antioxidant and anticancer activities. The analysis showed hydrazones (27.93%); phytosterols, namely, β-stesterol (11.09%); phenolic compounds; and finally chemical classes of chromene, furanone, and heterocyclic compounds to be the main responsible components. Although further studies are definitely required, it is evident that components currently regarded as waste have the potential to be utilized for therapeutic purposes.^39^

In another study including pineapple waste, phenolic yields of SSF when mixed with soy flour were studied.^40^ The treatment of equal amounts of pineapple residue and soy mixture (P5) yielded overall better antioxidant properties in comparison to the mixture containing 90% pineapple residue (P9). The phenolics content increased by 39.3% and 79.4% for P5 and P9, respectively. The results of the DPPH and β-carotene assays were similar, with the P5 samples being significantly higher than those of P9 in both cases. Similarly, Bind et al.^41^ conducted a study by using pomegranate peels mixed with soy flour and the microorganism *A. niger*. Daily variations of antioxidant production showed that TPC increased from 13.21 to 15.66 μg/mL, while DPPH increased from 16.88% to 43.01%, both showing their maximum results on day 4. However, when the media was optimized with both incubation time and pH having a value of 6, the results were 20.82 μg/mL for TPC and 46.21% for DPPH.

Grape pomace was subjected to SSF with the *Zygomycetes* fungi, *A. elegans* and *U. isabellina*.^42^ It was seen that the TPC content of the pomace fermented with *A. elegans* increased by 47% from the original value of 4.78 mg GAE/g dw by day 4, followed by a decrease until the end of the fermentation period; fermentation with *U. isabelline* resulted in a 27% decrease. A similar situation was observed for TFC with a 51% increase for *A. elegans* until day 4, followed by a decrease, and a 48% decrease for *U. isabelline* from the initial value of 0.96 mg QE/g dw. The increase of the *A. elegans* values was attributed to *A. elegans* being able to secrete cellulolytic enzymes thereby hydrolyzing β-glycosidic bonds and producing free phenolics. In contrast, the decline observed with *U. isabelline* could possibly be explained by the degradation and/or enzymatic polymerization of phenolic compounds. Antioxidant activity was measured using the DPPH radical scavenging assay and again showed opposing results for the two fungi. *A. elegans* fermentation showed an increase by 21.42% on day 4, which was followed by a decline. *U. isabelline* on the other hand first decreased antioxidant activity by 16% on day 8, followed by an increase.

Leite et al.^43^ used a variety of wastes obtained from the wine industry which included red and white grape marc, vine shoot trimmings, and grape stalks. The article stated that the results varied with different extracts and different strains used, with grape stalk having the highest TPC of 4.44 g/kg dw when water was used. The reason for this was attributed to grape stalk being removed at the beginning of the wine-making process and therefore subjected to minimum treatment resulting in the solid parts retaining the phenolic compounds. Grape marc, on the other hand, had lower TPC values in comparison as it was subjected to treatments such as decanting and distillation.

Black grape, apple, and yellow pitahaya residues were subjected to SSF with the microorganism *R. miehei* NRRL 5282 via two methods: freeze-drying and oven drying.^44^ The results were evaluated and compared for TPC and DPPH radical scavenging assay. The TPC results of the freeze-dried wastes were 1956, 477, and 495 mg GAE/100 g dw for grape, apple, and pitahaya, respectively. For oven-dried samples, the numbers were as follows for the same order of wastes: 1385, 362, and 615 mg GAE/100 g dw, respectively. The overall antioxidant activity showed a significant increase, and it was stated that the extracts which were enriched with phenolics would be a valuable source of natural antioxidants for the food industry.

Grape waste from a wine producer was used for SSF with *A. niger* GH1, PSH, Aa-20, and ESH.^45^ Although the results for all the strains provided higher results compared to their respective nonfermented samples, the *A. niger* GH1 strain exhibited the largest enhancement. The DPPH scavenging capacity was shown to be 90.8% for *A. niger* GH1, while the results of the other *Aspergillus* species were 81.4%, 83.3%, and 75% for PSH, ESH, and Aa-20, respectively. In terms of phenolic compounds, gallic acid content was evaluated. The highest increase in gallic acid was in *A. niger* GH1 with 9 mg/g, with the results of the others recorded as 6.7, 5.9, and 6.3 for PSH, Aa-20, and ESH, respectively. In a different study *A. niger* GH1 was used to aid in the SSF of locally obtained mango seeds.^6^ The TPC, DPPH scavenging activity, and overall antioxidant activity of the residue were elevated significantly with the TPC values rising from 984 to 3288 mg GAE/100 g.

A study conducted in Mexico used locally obtained fig byproducts from a jam and wine production company to compare the effects of four different fungal strains throughout SSF.^46^*R. oryzae* (PP4-UAMI), *Trichoderma* sp., *A. niger* HT4, and *A. niger* GH1 were used for the evaluation of TPC and antioxidant activity. Although all four cultures presented similar results, conditions such as mineral composition, pH, temperature, and moisture had to be optimized individually to achieve optimum yield. The highest TPC values were achieved for *A. niger* HT4 at 36 h and *A. niger* GH1 at 60 h. The TPC results showed a maximum of a 5.48-fold increase in comparison to the initial value, whereas this result was 98.54 for antioxidant activity.

Grapefruit byproducts moisturized at 50% and 70% were subjected to SSF by using Raimbault columns as bioreactors with *A. niger* GH1.^47^ DPPH and FRAP assays yielded similar results with both decreasing until 24 h and then increasing toward the end of the 120 h fermentation period for 70% moisture, while they increased stably for 50% moisture. Overall higher antioxidant values were observed for 70% moisture: DPPH activity was 2.15 times more than the initial value and measured at 7.64 mg/g dw at 96 h, while FRAP activity was 1.92 times more than the initial value and measured at 14.43 mg/g at 120 h.

Duff et al.^48^ studied the SSF of plum byproducts using the yeasts *A. niger* and *R. oligosporus*. Plum pomaces from a domestic grower along with brandy wastes from a local manufacturer were obtained for the experiment. The results showed an overall increase of total flavonoids and phenolics for both microorganisms, although the values of *R. oligosporus* were to some extent greater than those of *A. niger*. A similar case was observed for the total phenolic content, where the increases were over 21% for *A. niger* and over 30% for *R. oligosporus*. A distinct escalation was also observed in terms of antioxidant activity, measured using DPPH radical inhibition capacity, where the extracts fermented with *R. oligosporus* increased by 35.40%, and those fermented with *A. niger* increased by 27.70%. In another study, they investigated the effect of the same two fungal strains as above on apricot pomace. Total phenolics content showed an increase of 78% with *R. oligosporus* and 34% for *A. niger*. The same could be said for the total flavonoid levels which demonstrated an incline of 38% and 12% for *R. oligosporus* and *A. niger*, respectively. Both fungal fermentations provided an enhancement of the free radical scavenging capacities of methanolic extracts measured with the DPPH assay, and the antioxidant capacities were increased by more than 18% for both parties.^49^ The effect of fermentation on chokeberry pomace phenolics was analyzed using *A. niger* and *R. oligosporus*.^50^ The results were mainly in agreement with those of the two studies mentioned above. The values of the increase for *R. oligosporus* and *A. niger* were found to be 1.8- and 1.7-fold for total phenolics, 1.6- and 1.5-fold for total flavonoid, 1.5- and 1.35-fold for radical inhibition activity, 1.7- and 1.4-fold for antioxidant activity, and finally 1.2-fold for both strains for total anthocyanin content, respectively.

Vattem and Shetty^51^ used two nitrogen treatments, ammonium nitrate (NH_4_NO_3_) and fish protein hydrolysate (FPH), for the SSF of cranberry pomace with *R. oligosporus*. For both nitrogen sources, the β-glucosidase activity increased with a 60-fold increment for NH_4_NO_3_ and over 100-fold for FPH. A DPPH assay also showed an increase of 5% for ammonium nitrate; however, the increase of FPH was neglectable. An overall increase of total phenolics and antioxidant activity was also observed for the two treatments. The samples treated with NH_4_NO_3_ showed a 15% increase to 110 mg/10g dw, and those treated with FPH showed a 26% increase to 120 mg/10g dw. Finally, the β-carotene antioxidant protection factor increased by 20% and 25% for NH_4_NO_3_ and FPH, respectively.

Ajila et al.^52^ conducted a study using apple pomace to observe the changes in polyphenolics and antioxidant activity by SSF with the fungus *P. chrysosporium*. The polyphenolic compounds were extracted using ultrasonic extraction with either acetone or ethanol as solvents. A distinct increase in the extractable polyphenol content was observed, which in turn also resulted in the increase of antioxidant activity and free-radical scavenging activity. Furthermore, the study included the details of process optimization and stated that the amount of polyphenols that was able to be extracted varied depending on the type of solvent, temperature, time, and method. The results showed that the polyphenol content increased from 15.53 to 29.28 mg GAE/g dw in acetone extracts and from 11.28 to 22.71 mg GAE/g dw in ethanol extracts with both reaching their peak values on day 7.

Cocoa pod husk, cassava peel, and palm kernel cake, all of which are Nigerian agrowastes, were studied to examine changes occurring during SSF with the fungi *R. stolonifer* LAU 07.^53^ The results showed a general increase in antioxidant activity for all three of the waste products in DPPH assays. The IC_50_ (mg/mL) values of the methanolic extracts were also compared for fermented and unfermented samples. For palm kernel cake the value increased from 7.0 to 14.9, for cassava peel from 4.4 to 10.6, and for cocoa pod husk from 5.5 to 14.7 mg/mL. The study also investigated crude protein, crude fiber, ash, and lipid contents, all of which exhibited escalation. In another study, application of SSF on cocoa shells which are left over from cocoa processing was evaluated^54^ in terms of total phenolic compounds, reducing activity, and free radical scavenging activity. A notable increase in all three criteria was observed, with the fermented extracts exhibiting a 50–70% capacity to inhibit DPPH. However, in terms of total anthocyanin and flavonol contents, no changes were observed. The authors stated that solid-state fermented cocoa meal residue would be a viable antioxidant replacement for the food industry.

Maderia et al.^55^ used orange pomace, which can be found in abundance in Brazil due to the large orange juice industry, for the purpose of producing tannase and phytase enzymes through SSF while simultaneously measuring the antioxidant activity of the byproduct. They were successful in the production of both enzymes, and the antioxidant capacity showed a 10-fold increase when the microorganism *P. variotii* was used. The study however did go on to state that when the results of TPC were compared for before and after fermentation, the difference could be statistically neglected. This was attributed to the possibility that the phenolic compounds present in the orange pomace were transformed into lower-molecular-weight molecules which actually had higher antioxidant capacity throughout the process.

SSF was applied to a mixture of fruit and vegetable wastes consisting of orange, carrot, and papaya peels obtained from a local market with the use of *B. trispora* (+) MTCC 884.^56^ The purpose was to specifically produce β-carotene by the optimization of factors such as pH, temperature, nitrogen sources, and incubation time. The optimum parameters were stated as 96 h of fermentation at 30 °C and 6.2 pH. The results showed an increase in β-carotene and thereby antioxidant activity and stated that the compound was able to provide adequate scavenging effects for more than 90 days as shown by the DPPH assay. Acerola and guava byproducts were subjected to SmF over a period of 120 h using *Lactobacillus* isolates.^57^ The ascorbic acid contents of acerola declined throughout fermentation while the opposite was observed for guava. Guava had more acidic pH at the end of the fermentation in comparison to acerola, which could be the reason for the difference in ascorbic acid content as acidic pH values tend to inhibit ascorbic acid autoxidation. Total phenolic contents were observed to increase for both fruits throughout fermentation and were found to be 2669.811 and 60.62 mg GAE/100g for acerola and guava, respectively. ABTS and FRAP methods were used to measure antioxidant activity, where both showed an increase with the highest results being obtained at 120 h. ABTS results were 759 and 101 μmol TEAC/100 g, and FRAP results were 768 and 313.63 μmol TEAC/100 g for acerola and guava, respectively.

For the fermentation of blueberry pomace with *L. rhamnosus GG*, *L. plantarum*-1, *and L. plantarum*-2, TPC was shown to increase from 1066.89 to 4269.21 μg GAE/mL throughout the 28 h fermentation period, while TFC rose from 81.71 to 404.99 μg RE/mL.^58^ Both simulated gastric fluid and simulated intestinal fluid were used with differing pH conditions to study *in vitro* gastrointestinal digestion. A decrease was observed during both with the final results for simulated intestinal fluid being 4209.99, 4142.94, and 3838.62 μg GAE/mL at pH 1.5, 2.5, and 3.5, respectively. Simulated intestinal fluid results were lower with a value of 3190.77 μg GAE/mL at pH 7. This diminishment of TPC during *in vitro* digestion could be attributed to the binding reaction between protease and phenolic compounds. On the other hand, total anthocyanin content was increased almost 4-fold at pH 1.5 with a value of 19.20 mg/L, possibly due to their ability to endure acidic conditions.

Pomelo peels were fermented with four different alcohol concentrations: 1.5%, 4.5%, 7.4%, and 9.4%, using F33, a French active dry wine yeast.^59^ TFC, DPPH, FRAP, and ABTS^+^ radical scavenging activities were investigated at 3 stages: before fermentation, after fermentation, and postfermentation. While TFC increased after fermentation for all 4 concentrations, the maximum values were obtained for postfermentation of the 9.4% concentration at 223.96 mg RE/L. According to the results of the DPPH assay, there was an increase with the first two concentrations after fermentation with their highest values being 32.16% and 34.77% for 1.5% and 4.5% concentrations, respectively. 7.4% and 9.4% concentrations showed a constant increase, where they had their highest values being obtained for postfermentation at 39.11% and 34.33%, respectively. Moreover, ABTS^+^ radical scavenging activity decreased during fermentation and then increased slightly during postfermentation. The highest results obtained were for a 9.4% concentration, with prefermentation and postfermentation being 84.03% and 79.54%, respectively. FRAP values, expressed as ferrous sulfate (FeSO_4_) concentration in mmol/L, were seen to decrease for the 1.5% concentration; however, they increased for the other concentrations. Overall, it was seen that the highest concentration of alcohol was able to provide the highest antioxidant activity during the fermentation period.

As can be seen with the studies detailed above, the phenolic compounds and antioxidant activity results obtained can vary with the byproduct used, fermentation type, and conditions such as time, pH and temperature, assays applied to observe the said properties, the prior treatments the wastes were subjected to, extraction types, as well as the country of origin of the byproduct. Nevertheless, utilizing fermentation techniques to valorize byproducts of fruits has favorable outcomes and has the potential to be valorized for further use in the food industry upon optimization of fermentation, extraction, and application techniques.

## 4 Commercialized Applications and Consumer Acceptance

The process of obtaining compounds with antioxidant properties from food wastes and byproducts is a work in progress; however, the studies conducted in this regard showcase the many benefits. Furthermore, additional studies and applications are being researched every day in order to create more feasible and sustainable processes which would be advantageous to use on an industrial scale.^60^ However, the valorization and commercialized application of the mentioned compounds alone will not be sufficient if the products are to succeed in the market. Consumer acceptance is also an incredibly important factor to take into consideration.

Some of the patented methodologies and product-specific applications of food waste sources are citrus peels, cheese whey, olive mill waste, tomato waste, soy protein isolate wastewater, shrimp and crab shell, depectinated apple pomace, grape and cranberry seed, pomegranate rind, and seedcase residues.^61^ These have been used for a variety of food supplements and additives, with some extracts even being used in the cosmetics industry. In a study conducted by Lavecchia and Zuorro,^62^ cell-wall degrading enzymes were used to extract lycopene from tomato peels, and the results showed that a complete recovery of the natural antioxidant was possible given the optimum processing conditions. In 2010, they received their patent for the extraction of lycopene using a solvent mixture and stated that the industry of natural extracts strongly needs this product. Another potential application of the phenolic compounds attained through SSF is their use as edible coatings and films.^63^ These antioxidants are of high quality and stability and can extend the shelf life of foods as well as meet consumer demands regarding the use of natural additives. The authors note that their use instead of traditional packaging materials is an option worth investigating further.

Research and surveys conducted in regards to the consumer acceptance of additives obtained from food waste and byproducts are insufficient. It is possible to gain an idea of where the public mainly stands in respect to this topic through some waste recovery studies which include consumer feedback on specific products or components. However, in order to gain an in-depth understanding of how the customers who will purchase products containing these compounds feel and think about the subject, more broad surveys and studies must be organized. Along with general questionnaires, additive-, waste-, and end product-specific surveys should also be studied.

## 5 Conclusion

It is evident that food waste is a big concern for both the environment and humans. The most important step to take in solving this problem is to reduce the amount of this depletion through education; use of high-quality equipment; appropriate storage, transportation, and distribution conditions; as well as creating awareness to the consumer. Even through these applications, the unfortunate reality is that it is still impossible to completely eliminate food waste. Furthermore, there are exceptional amounts of byproducts produced during the processing of fruits and vegetables, which are seldom used. These byproducts contain various classes of components that have many varying health benefits as detailed above. Considering that many antioxidant additives used in the food and pharmaceutical industries are produced synthetically, it could be considered wise to take advantage of these byproducts and consumable wastes. Moreover, customers conscious of the matter may be more drawn to purchasing products with natural additives in comparison to the synthetic ones.

The studies mentioned in this review show that the fermentation of various food wastes exhibits increased antioxidant activity. As antioxidant activity is a broad term, compounds exerting antioxidant properties are high in number and variety. Accordingly, the antioxidant properties of a food product can be measured by numerous methods, and there is an abundance of studies in the literature regarding their enhancement through fermentation. In many cases, optimizations of parameters such as pH, temperature, water activity, and incubation times were conducted. Furthermore, a variety of food wastes have been studied extensively, and a majority of the research results are in accordance with the statement that fermentation is able to improve and enhance antioxidant properties. In addition, studies in which nonwaste food materials were subjected to fermentation were higher in number and yielded similar results, although they were not included in this review.

The mechanisms behind the interaction of fermentation and antioxidative compounds also fluctuate, and although this area does require more specific research, the results show that the correlation is positive. By using suitable and safe extraction methods, many products regarded as waste can be valorized to be used in not only the food industry but pharmaceutical and cosmetic industries as well. Although there are a number of laboratory-scale studies conducted in this area, their large-scale applications are restricted and insufficient. Further *in vitro* and *in vivo* studies are required to obtain more determinative information regarding the bioaccessibility and bioavailability of the aforesaid compounds. There is also a large amount of studies comparing SmF and SSF available in the literature. As mentioned in detail above, both processes contain a set of both advantages and disadvantages, and the selection of which process is the most suitable is a multisided choice. The adaptation difficulty of SSF to a large-scale process and its nonuniformity in terms of heat transfer are two of the main areas which need to be investigated further. Furthermore, it has been found rather difficult to compare SSF and SmF in a determinative manner as the methods themselves require different processing conditions in order to achieve maximum yield.

It is important to establish the correct understanding when it comes to valorizing and reusing, especially regarding food products. Consumer acceptance is one of the important, if not the most important, factors to take into consideration. The response to natural additives is almost always positive; however, the source of these natural antioxidants being wastes may present an acceptance problem. As the public is not informed about the specifics of the processes conducted to enhance, improve, and extract antioxidants from byproducts and food wastes, the feedback can be negative. Countries need to focus on their largest wastes and byproducts along with the most feasible final product to use these waste-gained compounds in. This will allow them to optimize recovery procedures accordingly and ensure the utmost suitability for the market. Although an industrial-scale SSF setup does have its disadvantages, when weighed with the overall advantages, it proves to be a process worth investing in. The economic, environmental, and social impacts of a system which produces natural antioxidants, while simultaneously tackling the waste problem, would be a huge asset not only to the individual countries but also on a global level. Evidently, further research and pilot plans are required to carry out these processes in the industry, and succeeding in doing so seems to be a promising method of obtaining value-added wastes thereby achieving sustainable food production and maximizing profits in the long run.

Although there is a substantial amount of research conducted in respect to the effects of fermentation on various food products, those which are applied on wastes and byproducts are quite limited in comparison. In light of the collective information which can be gained from published research regarding the antioxidant properties of various fruits, those which focus on the fermentation of food products and extraction of antioxidants for the purpose of utilization in other food products, it is possible to adapt and design waste- and product-specific fermentation processes.
